# Process evaluation of an integrated community-based intervention to improve family planning, sexual reproductive Health, and wellbeing among Syrian refugee women and girls in Lebanon during active conflict

**DOI:** 10.1186/s13031-026-00748-7

**Published:** 2026-01-17

**Authors:** Shadi Saleh, Hady Naal, Asmaa El Dakdouki, Zahraa Chamseddine, Veloshnee Govender, Dalia Sarieddine, Gladys Honein AbouHaidar, Tania Bosqui, Hani Tamim, Fouad Fouad, Sara Ibrahim, Zahi Abdul Sater, Rachel Vanderkruik, Lale Say

**Affiliations:** 1 Global Health Institute, American University of Beirut, Beirut, Lebanon; 2 Faculty of Health Sciences, American University of Beirut, Beirut, Lebanon; 3 Department of Sexual and Reproductive Health at the World Health Organization, Geneva, Switzerland; 4 Hariri School of Nursing at the American University of Beirut, Beirut, Lebanon; 5 Department of Psychology, American University of Beirut, Beirut, Lebanon; 6 Trinity Center for Global Health, Trinity College Dublin, Dublin, Ireland; 7 Department of Internal Medicine, Clinical Research Institute, American University of Beirut, Beirut, Lebanon; 8 College of Medicine, Alfaisal University, Riyadh, Saudi Arabia; 9 Department of International Public Health, Liverpool School of Tropical Medicine, Liverpool, UK; 10 Department of Health Policy and Management, American University of Beirut, Beirut, Lebanon; 11 Massachusetts General Hospital, Ammon-Pinizzotto Center for Women’s Mental Health, Harvard Medical School, Boston, MA USA

**Keywords:** Process evaluation, Refugee, Community-based trial, Lebanon, Conflict

## Abstract

**Background:**

This study presents the first process evaluation of an integrated family planning, sexual reproductive health, and wellbeing community-based intervention among Syrian refugee women and girls in Lebanon. This intervention, known as the Self-Efficacy and Knowledge (SEEK) intervention, was developed by the World Health Organization as a low-resource and low-intensity initiative, and led by trained paraprofessionals (community health workers).

**Methods:**

The intervention was implemented between September and December 2024, a period marked by active conflict in Lebanon. A mixed-methods process evaluation was conducted, triangulating data from satisfaction surveys, field observations, and semi-structured interviews with participants, health workers, and program staff. Quantitative data were analyzed using SPSS, and qualitative data were analyzed using qualitative content analysis. Data collection tools assessed satisfaction, feasibility, fidelity to content, logistical and contextual barriers.

**Results:**

The evaluation revealed high participant satisfaction, with over 90% of participants rating session quality as good or excellent. Participants valued the program’s relevance, paraprofessionals community alignment, and the inclusion of interactive and visual aids. Paraprofessionals expressed satisfaction with the training and delivery process but, along with attending psychologists and midwives, reported the need for more soft-skills training and presentation skills. Logistical challenges included child care needs, transportation barriers, and the necessity of flexible scheduling. The war on Lebanon posed major implementation hurdles, requiring adaptive strategies such as remote coordination and increased reliance on leadership of local staff. Cultural and gender norms affected engagement, particularly around SRH content, with participants recommending greater involvement of men and household decision-makers. The presence of local women committees, research assistants, and field coordinators was key to maintaining trust, communication, and retention of participants.

**Conclusions:**

This evaluation demonstrates that SEEK is feasible, acceptable, and adaptable even in the context of active conflict. Its community-led design supported engagement and delivery, underscoring the importance of flexible and locally grounded implementation strategies in fragile settings.

**Trial registration:**

Retrospectively registered on NIH clinical trials reference# NCT07008950.

## Introduction

 The Eastern Mediterranean Region (EMR) has increasingly experienced humanitarian crises over decades, leading to ongoing continuous instability and complex challenges. Over half of the world’s displaced populations originate from the region, with an estimated 62 million individuals experiencing significant health needs [1]. Among the most affected are women and adolescent girls, who face heightened health risks due to inadequate access to vital services [2, 3]. In particular, access to Sexual and Reproductive Health (SRH) and Family Planning (FP) services remains severely restricted in humanitarian settings, leading to poor SRH and wellbeing outcomes [4–7]. These include unwanted pregnancies, unsafe abortions, Sexually Transmitted Infections (STIs), and delivery complications, among others [6, 8, 9]. Some of the main barriers to SRH access include limited knowledge, persistent gender stereotypes and cultural stigma, and restricted availability of services [10]. Additionally, prolonged conflict has significantly impacted well-being and mental health, especially among women, due to sociocultural factors, violence, displacement, and poverty [1, 11, 12].

The protracted Syrian crisis serves as a stark example of this ongoing regional crisis, with approximately 6.4 million refugees displaced globally [13, 14]. Lebanon, which hosts the highest per capita influx of refugees, has taken in over one and a half million Syrian refugees, which amounts to approximately 25% of its population [13, 15, 16]. Within this refugee population, 16% are aged 15–24, with females constituting the majority [17, 18]. Many reside in overcrowded informal tented settlements and substandard non-residential structures, particularly in the Beqaa governorate [19, 20]. Previous need assessments have identified women’s health and mental health as key priorities among Syrian refugees in Lebanon, with barriers to access including limited geographical availability and low health awareness [16]. Healthcare provision for this population involves coordinated efforts by the Ministry of Public Health (MoPH), United Nations High Commissioner for Refugees (UNHCR), and Non-Governmental Organizations (NGOs) [21]. However, refugees frequently encounter financial, administrative, and legal challenges when seeking care. Public health services tend to not be free-of-charge, and many refugees lack medical records required for treatment [22]. The Lebanese healthcare system operates predominately through the private sector [23] and UNHCR offers only partial financial support. Many medical cases are excluded or partially covered, making the costs prohibitive for most refugees [24]. Compounding these concerns, nearly 80% of Syrian refugees in Lebanon lack legal residency, which limits their access to healthcare because [25] identification is often required to receive care, creating further access barriers for those without documentation [26]. These intersecting constraints are especially severe for women and girls, who may face additional risks due to mobility restrictions [27, 28], increased exposure to Gender-Based Violence (GBV) [29–31], and associated health consequences such as high risk of miscarriage and unsuccessful conception [32]. The situation has been further compounded by the recent conflicts in Lebanon and the region in late 2024. This period, especially during Lebanon’s active conflict spanning from September and December 2024, represented the most severe conflict since the 2006 war [20], resulting in the displacement of more than one million people, including displaced Syrian refugees [33, 34].

This recent conflict has significantly impacted the mental health of the displaced population, particularly those who have experienced death or separation from family members [35]. Poor mental health is associated with reduced help-seeking behaviors and may lead to risky behaviors, such as inadequate use of contraception, negatively affecting SRH outcomes [36, 37]. Factors related to psychosocial vulnerability (e.g. social isolation, lack of family support, self-neglect, diminished motivation, cognitive impairment among others) combined with socio-economic disadvantages may contribute to adverse health behaviors such as substance use, and unprotected sex among others [38]. Consequently, mental health interventions are essential to enhance SRH outcomes in humanitarian settings [39].While prior studies differ in populations and outcomes targeted, several offer conceptually relevant insights approaches. For instance, interventions that combined mindfulness and participatory discussions on reproductive health topicshave been shown to improve knowledge and mindfulness among adolescents with parental HIV [40]. The PartnerPlus intervention, demonstrated that psychoeducation (e.g., cognitive-behavioral skill communication, sexual negotiation, conflict resolution), when paired with SRH education (e, g., STI/HIV prevention, and use of male and female condoms) contribute to increased STI knowledge and improved negotiation skills [41]. Additionally, a program in India targeting adolescent girls integrated psychosocial skills (e.g., emotion regulation, assertiveness, communication skills, and problem-solving) with SRH education, resulting in increased SRH knowledge, and more equitable gender attitudes [42]. While these examples differ in contexts, they showed that integrated psychosocial support with SRH education can improve health knowledge, behavior, and attitudes. This supports the rationale for integrated approaches targeting both psychosocial wellbeing and SRH, particularly in crisis-affected and underserved settings.

In humanitarian settings, where health workforce shortages and service disruptions are common, community-supported interventions have emerged as potentially effective strategies to support sustained and accessible health service delivery [43–46]. However, little evidence exists on implementing community-based approaches including psychosocial support aiming to improve access to SRH services.

Despite the significant SRH and mental health needs in humanitarian settings, and the well-established link between them, integrated interventions addressing both remain limited [1, 39, 47]. Research suggests that combining psychosocial support (PSS) with SRH intervention can enhance access to quality care and support better overall well-being [48, 49]. Building on this evidence base, the World Health Organization (WHO) developed an integrated PSS-SRH intervention package that is low intensity and low resource, to support adolescent girls and young women’s access to SRH services in humanitarian settings [10].

The Global Health Institute (GHI) at the American University of Beirut, in collaboration with the Special Programme of Human Reproduction (HRP) within WHO’s Department of Sexual and Reproductive Health, evaluated the implementation of this package focusing on married adolescent girls and young women refugees aged 15–24 in Lebanon’s Beqaa governorate. Details about the findings of the RCT have been reported in other manuscripts that are currently under review, however a summary of their findings can be found in the supplementary material for this present manuscript.

The study aimed to evaluate [1] the effectiveness of SEEK package in improving access to SRH and FP services and wellbeing, through an outcome evaluation, and [2] its applicability, feasibility, and acceptability through a process evaluation. This present article reports findings of the process evaluation.

## Methods

### Study design

**Fig. 1:**
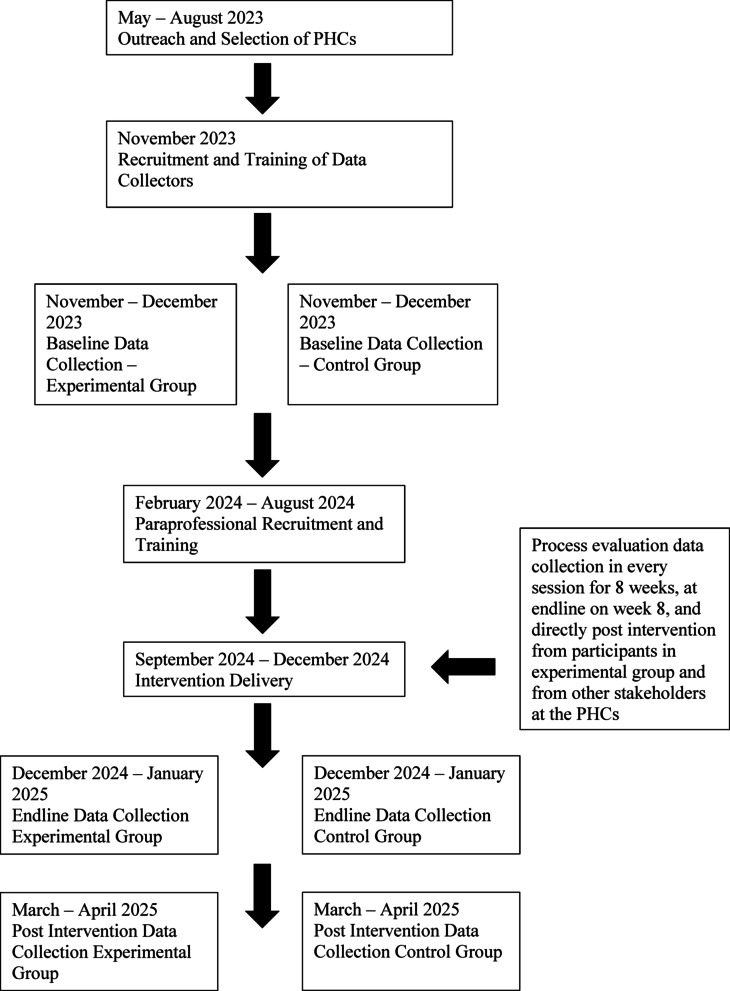
Flow diagram of key study phases. This process evaluation study was conducted under a larger parent trial which aimed to evaluate the SEEK intervention through a community-based randomized controlled study design. It was retrospectively registered on clinicaltrials.org under reference number # NCT07008950

The present study was conducted under a larger parent study following a community-based randomized controlled trial design that was retrospectively registered on clinicaltrials.org reference number #NCT07008950. At the time of writing this manuscript, further information about the study design is reported in manuscripts that currently under review elsewhere. However, in general, the trial aimed to evaluate the SEEK intervention package, which examined changes in family planning uptake as a primary outcome, and changes in wellbeing and psychosocial factors as secondary outcomes. Additional details about the trial and the SEEK intervention are discussed in subsequent sections in this present manuscript. As part of the larger study, this process evaluation was conducted, and it adopted a mixed-methods approach, collecting qualitative and quantitative data from multiple sources of stakeholders involved directly or indirectly with this intervention across time. For instance, satisfaction surveys were collected post every session from participants enrolled in the study, whereas by the end of the intervention, multiple staff were interviewed through in-depth key informant interviews such as paraprofessionals, attending psychologists and midwives, along with the nurses and managers at the targeted Primary Healthcare Centers (PHCs) (please see Fig. 1 and subsequent sections below for more details about the methodology and study phases).

### The WHO PSS-SRH integrated intervention package

The Self-Efficacy and Knowledge (SEEK) package was designed to enhance FP, SRH, and wellbeing in humanitarian settings through an integrated approach. It is a low-intensity/low-resource psychosocial support – SRH integrated intervention package composed of eight modules (see Table 1) focusing on SRH education, psychoeducation, and skills acquisition in domains such as emotional regulation, communication problem management, decision-making, and self-efficacy [10]. The package was delivered by community-based paraprofessionals who have completed at least twelve years of formal education and have undergone robust training by mental health and SRH experts. Additionally, the intervention was structured as a weekly 90-minute session over eight weeks. Content was delivered in Arabic, through several modalities including PowerPoint presentations, interactive lectures, discussions, case studies, contextualized practical activities, and homework assignments. The target population comprised adolescent girls and young women refugees aged 15–24 in humanitarian settings.

The intervention development underwent multiple rounds of revisions based on consultative processes before conducting this evaluation. It was also piloted in Lebanon before formalizing it as an intervention package.

**Table 1:** Sessions overview

Session number	Content covered
Session 1: Introduction and Emotion Regulation Skills	-Introducing the participants to one another and providing an overview of the program. -Establishing group guidelines to create a safe environment. -Education on recognizing emotions and articulating feelings. -Teaching grounding exercise for managing intense emotions and distress.
Session 2: Linking Actions and Feelings	-Engaging participants in discussion regarding their personal strengths and how these strengths might be related to SRH. -Education on the role of social support and self-care in enhancing feelings and providing strategies to strengthen social support.
Session 3: Communication and Assertiveness Skills	-Introducing effective communication to learn different types of communication and differentiate between them. -Providing information on accessible health services and using assertiveness to overcome barriers to healthcare access.
Session 4: Family Planning and Contraception	-Education about reproductive systems and building sexual self-efficacy. -Understanding effective contraceptive methods and distinguishing between facts and myths. -Building communication skills with family members.
Session 5: Contraception for Health and Problem-Solving Skills	-Learning about STIs and protection methods. -Developing problem-solving skills and applying it to SRH concerns.
Session 6: Problem-Solving and Gender Norms	-Practicing problem-solving skills for health protection. -Understanding gender norms, healthy and unhealthy relationships and violence types.
Session 7: Consolidating Skills and Contraception Action Planning	-Adopting assertive communication techniques to handle conflicts in relationships, and reinforcing skills learned from previous sessions. -Learning about the pressure line and how to negotiate. -Use of contraception and steps for contraception action planning.
Session 8: Skills Practice and Closing	-Learning to create an action plan for contraception and reinforcing skills acquired from previous sessions.

### Study settings selection

GHI collaborated with the Ministry of Public Health (MoPH) to obtain the list of PHCs in the Beqaa governorate that were recommended as a potential study site, due to the region’s high concentration of Syrian refugees.

Subsequently, the team conducted thorough field visits and outreach to these PHCs, during which two were selected based on the following criteria:

- Operates in Beqaa governorate.
- Provides SRH services to female Syrian refugees in a crowded area.
- Able to refer refugees with possible health problems (STIs, mental health) to relevant health centers with appropriate follow-up.
- Includes at least two clinical spaces to perform gynecological examinations.
- Able to provide the space needed for all phases of the study.

### Study phases

**Phase One**:

#### 1. Recruitment of participants (Pre-health days)

In this section, participants refer to women and girls from affected communities who were enrolled in the study. Eligibility criteria further required that participants be married, not breastfeeding, not pregnant, not suffering from a chronic disease that might hinder adherence to the study, not undergoing specialist mental health treatment, and willing to be part of the study. To identify potential participants, the team coordinated with the heads of the selected PHCs and requested that they organize a list of eligible candidates from their databases. Subsequently, a staff member from each PHC contacted these individuals by phone using an invitation script and invited them to attend a series of health days held between November and December 2023. Health days were organized to conduct necessary screenings for eligibility and baseline data collection at the two selected PHCs. For individuals aged 18 and above, staff reached out to them directly, while for those under 18, their legal guardians were contacted. Additionally, all contacted individuals were requested to bring their personal ID for identity verification, and minors were required to be accompanied by two legal guardians.

#### 2. Recruitment and training of data collectors

Fifteen data collectors were recruited and participated in a two-day training session held on November 10 and 13, 2023, at the municipality of Anjar in the Beqaa governorate. The first day provided an overview of the SEEK study, covered informed consent procedures, and introduced the baseline questionnaire with an emphasis on handling sensitive topics. The training also highlighted the importance of non-assumptive questioning and discussed ethical considerations related to confidentiality, beneficence, and risk assessment. The second day reinforced these skills through role-playing activities designed to evaluate data collectors’ competence, beginning with a brief refresher of the previous day’s material.

#### 3. Recruitment of local women community

The study involved recruitment of Local Women’s Committees (LWC) to promote a supportive environment for the participants in the study and to mediate communication between the research team, paraprofessionals, and participants. To enroll LWC members, the research team first requested that the selected PHCs provide a list of Syrian refugee women with at least primary or intermediate levels of education, ensuring they were literate. The team then contacted these potential candidates, and eight LWC members were recruited, four from each PHC. Two were needed per PHC, with the others serving as backups. The presence of LWC members not only fostered trust but also ensured a culturally sensitive approach during the intervention, thereby promoting a sense of community among participants and facilitating the sharing of thoughts and experiences.

#### 4. Health Days

A total of nineteen health days were conducted, with ten days at one PHC and nine days at the other. Two selected LWC members were present at each center to ensure effective management; these members played a crucial role in managing participant flow and addressing concerns. Moreover, RAs managed scheduling and ensured participants arrived on time, while one coordinator was located at each center to oversee the overall process. Participants were divided into morning and afternoon groups to avoid overcrowding.

Participants were provided with transportation incentives, hygiene kits, and an optional OBGYN examination. The OBGYN exam was an optional consultation that they could benefit from or refuse. It consisted of a standardized consultation that did not focus on any type of screening in particular. Each participant was assigned a unique code instead of using their name to ensure anonymity and confidentiality. After data collection, a statistician conducted randomization to evenly distribute all participants screened to be eligible for enrollment in the intervention into experimental and control groups. All women and girls who were randomized into the SEEK intervention participated in the health days.

**Phase Two**:

#### 1. Recruitment and training of paraprofessionals

To effectively conduct the intervention, twelve Syrian women from the community were recruited as paraprofessionals based on suggestions from the selected PHCs. In this study, the term paraprofessional is synonymous with community health workers or community leaders. It refers to individuals from affected communities who do not have professional credentials but who have been prepared to provide basic services and health promotion to their communities through training and capacity building. They underwent a twenty-day training program, initially planned as twenty consecutive sessions but adjusted due to delays in obtaining approvals from AUB and WHO Institutional Review Boards (IRB) for the second phase, which included the intervention delivery, endline, and post-3-month data collection. Consequently, the training was divided into two parts: the first fifteen days from February 2024 till March 2024 and five booster sessions held between April 2024 and August 2024. This gap allowed for periodic refresher leading up to the intervention commencement, supplemented by follow-up phone calls to ensure preparedness and address any questions.

The training was conducted by experts in SRH and mental health, selected for their experience working with the Syrian refugee population and their proficiency in delivering such trainings. This program equipped the paraprofessionals with the necessary skills and knowledge to deliver the intervention effectively and address potential concerns during the sessions. Specifically, it included activities led by experts on facilitation competencies, roles during the interventions, and strategies for handling challenging scenarios. Additionally, an overview of MH and SRH was provided, followed by detailed content delivery to enable paraprofessionals to conduct sessions independently.

Role plays and discussions were an integral component of the training. The five booster days focused primarily on role plays, requiring paraprofessionals to prepare and deliver an entire session to their colleagues, simulating the intervention environment. While some paraprofessionals initially displayed discomfort discussing SRH and MH topics, over time, they engaged more openly, sharing personal experiences and addressing societal taboos within their communities. Notably, paraprofessionals with previous experience in similar roles exhibited stronger performance, facilitating their adaptation to the training. Of the twelve recruited paraprofessionals, only seven completed the training program due to personal and logistical challenges. Initially, six paraprofessionals were to be selected for the intervention, with four others designated as backups. However, based on experts and research teams’ feedback, only four paraprofessionals demonstrated the ability to lead future sessions based on subjective ratings on technical knowledge, fidelity to content, and others as assessed by training supervisors and subject matter experts.

To further support the execution of the intervention, two midwives, two psychologists, and the eight selected LWC members attended the training. Recommended by the selected PHCs, these individuals participated to familiarize themselves with the content and foster cohesive team dynamics. Their role encompassed attending all intervention sessions to assess fidelity to content, supporting paraprofessionals, and managing any emerging MH and SRH issues.

#### 2. Intervention delivery

In the pre-intervention phase, the experimental groups were contacted and organized into sixteen groups — eight per center —with each group comprising twelve participants. Each center hosted two groups daily (morning and afternoon sessions), for a total of eight groups each week per center. Over the period of eight weeks, each group received one session per week, totaling eight sessions. The intervention started in September 2024. Two paraprofessionals were assigned to each center, with each paraprofessional being responsible for delivering sessions to the same group throughout the intervention. This consistent assignment allowed paraprofessionals to establish relationships with participants, creating a comfortable space for them. The intended group size of twelve participants was not always reached; some groups included one or two more or fewer participants. There were significant reallocations between groups, and some participants missed sessions. Participants who were unable to attend their allocated sessions were rescheduled so that they may attend with another group. If they skipped the rescheduled session, they received a recap at the beginning of the next session. Additionally, some participants tended to change their groups due to the inconvenient scheduled time or to attend with a friend.

**Table 2:** Participant attendance sheet

Session	Attended	No-Show	Dropouts
**1**	171	4	0
**2**	161	11	3
**3**	131	29	12
**4**	122	30	8
**5**	126	10	16
**6**	123	6	7
**7**	122	7	0
**8**	122	4	3

Throughout the course of eight sessions, participant attendance was documented (see Table 2). A total of 175 participants were enrolled in the intervention arm. At baseline, the mean age of participants was 20.86 years, with a mean age at marriage of 16.36 ± 2.04 years. The majority (79.4%) had an education level of intermediate and below, and only 18.3% were currently working. Attendance varied, with one hundred and seventy-one participants attending the first session and progressively decreasing in subsequent sessions. Dropout’ rates and non-attendance changed during the course of the sessions. The highest attendance was reported in session one (171 participants), while the lowest was in sessions four, seven, and eight (122 participants). Dropouts were reported in all but two sessions, with session five having the highest dropout rate (16 participants).

Dropouts and missing sessions occurred for several reasons, including war-related concerns, family refusal, work, loss of identification documents, missed follow-up calls, relocation, illness, giving birth, or childcare responsibilities. Many participants brought their children to sessions, which caused some distractions to attending participants as the venues did not include space and staff dedicated for children. The initial protocol stated that if a participant missed session one, they would be automatically moved to another group to attend session one. If they completely missed session one, they would proceed to session two. Missing one session allowed continued participation, but missing two sessions (except session one) resulted in being considered a dropout. However, these rules were modified after week two and the first day of week three, when a war escalated in Lebanon, which necessitated around one-month pause to develop a mitigation plan.

During this period, the research team contacted participants and local teams, including paraprofessionals, LWCs, midwives, and psychologists, to ensure they could access the PHCs safely. As part of the modifications and flexibilities introduced, participants were allowed to miss up to three sessions before being considered dropouts. Given the Beirut-based team’s inability to travel to Beqaa governorate, two field coordinators from Beqaa were recruited, trained online, and assigned to oversee and manage the intervention locally. The Beirut research team coordinated with them remotely to ensure smooth delivery and implementation. All necessary materials were provided to the Beqaa team. Several dropouts were faced due to participants returning to Syria or being internally displaced from Beqaa to other governorates. Despite these challenges, the intervention continued and concluded in December 2024. Throughout the intervention, we maintained regular phone communication to remind participants to attend sessions. However, reaching participants was sometimes challenging if the phone was often owned not by the participant but by their husband or relative, who might forget to relay the message. Moreover, some phone numbers were out of service or had changed owners.

Women and girls who were randomized into the non-intervention group only completed the baseline, endline, and 3-months post survey, and received reimbursements for their participation. Later on, by the end of the study period, they received the same material that the intervention group received.

#### 3. Process evaluation measures

A process evaluation was carried out to assess the satisfaction, content fidelity, attrition, accessibility, feasibility, and overall impact of the SEEK intervention. This was conducted through a mixed-methods approach, employing both quantitative and qualitative tools. Each tool was tailored to assess different program aspects, as shown below:

**Table:** 

Tool	Description
*Paraprofessional package satisfaction survey*	assessed the satisfaction of the four paraprofessionals who facilitated the intervention package regarding their ability to deliver it. The survey was filled out by these paraprofessionals at the end of the intervention. It included Likert scale questions to self-evaluate the paraprofessional’s quality of implementation across all sessions (ranging from superior to poor), with paraprofessionals elaborating on the reasons behind their ratings. In addition, open-ended questions explored the most appreciated aspects of the SEEK program and its paraprofessional training, among others.
*Participant session satisfaction survey*	evaluated overall satisfaction of participants at the end of each session. It was completed by the participants and included Likert scale questions assessing the quality of the session. It also includes one open-ended question to gather any additional feedback about the attended session. This tool was administered by study staff after every session.
*Participant package satisfaction survey*	evaluated overall acceptability, satisfaction, feasibility, and content fidelity by participants at the end of the last intervention session. It included Likert scale questions assessing the quality of services, the extent to which the SEEK program met participants’ needs, satisfaction with the support received, its effectiveness in managing their concerns, and whether they would recommend or consider a similar intervention. The survey also included seven open-ended questions exploring participants’ satisfaction with the overall package.
*Semi-structured interview*	assessed the additional challenges and perceived benefits of the package, as well as to inform the overall impact of the intervention, semi-structured interviews were conducted with various stakeholders. The interviewees included two paraprofessionals, two members of LWCs, two center managers, two PHC nurses, and six participants. The semi-structured interviews were conducted one month after the intervention concluded through phone calls.
*Participant attendance sheet*	assessed the attrition rate after each session. It was completed by field coordinators managing in-session logistics.
*Paraprofessional competence assessment*	evaluated the general competence of the paraprofessionals delivering the intervention after each session. This survey was filled out by the attending psychologists and midwives and included Likert scale questions (ranging from superior to poor), followed by open-ended for comments on each rating.
*Field Observations*	Field observations were reported by the field research team to provide insights into the practical implementation of the intervention. It allowed the team to identify contextual factors influencing outcomes and uncover unexpected challenges or successes no targeted by the aforementioned tools. This method included direct observations of sessions, paraprofessional-participant interactions, and the overall environment in which the intervention occurs.

### Data analysis

SPSS was used to analyze quantitative data reported as descriptive and cross-tabulated statistics. For qualitative data obtained from open-ended questions, we followed qualitative content analysis technique to code variables, identify emerging themes, and to report them under larger categories. Semi-structured interviews were audio-recorded and transcribed verbatim into Arabic by the research team and then translated to English to maintain the nuances of participants’ responses. The identified patterns were discussed among the research team to reach a consensus whereby qualitative and quantitative data were finally triangulated to reach convergence across data sources. All members of the research team have previous experience working with refugee women and are well familiar with the context of Syrian refugees in Lebanon.

### Ethical approval

Ethical approval was obtained from the Institutional Review Board (IRB) at the American University of Beirut (AUB) and at the Ethics Research Committee (ERC) of World Health Organization (WHO). Before data collection, all participants provided informed consent, ensuring they understood the objectives, procedures, potential risks, and benefits of the study. Furthermore, the intervention manual underwent inspection by relevant ethics committee boards, and staff underwent rigorous approved trainings to ensure ethical best practices.

## Results

The results section synthesizes key aspects of the process evaluation, after triangulating all collected qualitative and quantitative data, into the following themes [1] participant satisfaction [2], paraprofessional performance and satisfaction [3], logistical considerations [4], contextual considerations [5], content considerations [6], cultural and gender considerations, and [7] field observations.

### Participant satisfaction

When reviewed holistically, post-session participant satisfaction data revealed high satisfaction across all sessions, with over 90% of respondents rating session quality as good or excellent, and believing that sessions can help them effectively manage their problems (see Table 3). Indeed, this was confirmed by participant interviews as one of them mentioned that:

“*we benefited from the project and learned things we did not know before*,* like STIs*” and “*the topics raised were new to us and we were not aware of them previously*” – Participant from experimental group.

Collectively, across all sessions, reports of feeling positive emotions and gaining new knowledge and skills useful for personal development were found in the open-ended section of the satisfaction survey. However, some challenges were reported such as distractions caused by presence of children during some sessions, finding the paraprofessional unclear or unengaging, and wishing for some sessions to be longer in duration (see Table 4).

Similarly, the package satisfaction survey administered at endline revealed high satisfaction with over 80% satisfaction reported on all items associated with the overall package, setting of implementation, facilitator performance (see Table 5).

As for the open-ended questions, findings suggest that the primary motivation to attend the program is to improve knowledge and to receive incentives (monetary compensation, food, contact with healthcare professionals, among others). This was also echoed by the PHC head who mentioned that:

“*the more you offer [financially] the more attendance increases… Some people come to register just for that*,* even though the topic doesn’t concern them*,* while others tell you the topic is important to them. Before thinking about education*,* they are considering what they will receive materially in terms of incentives*,* and it’s not wrong – if you offer something small it can motivate them more*” – Head of PHC.

When asked about suggestions to improve SEEK program, participants reported that the program can benefit from (1) better planning for session length, (2) provision of more financial incentives, (3) provision of staff and space for children, (4) optional access to private follow-up session especially for sensitive or more complex topics, (5) increasing training for paraprofessionals especially on soft skills such as communication and presentation skills, and (6) use of interactive modalities such as video and graphic tools to account for participants who might be illiterate.

### Paraprofessional performance & satisfaction

For the most part, paraprofessional reported satisfaction with delivering the program, its content, its structure, and with the preparatory training they received.

“*The gradual progression of topics in the [SEEK] manual was one of the key factors that facilitated the successful implementation of the project” - Paraprofessional*.

Paraprofessionals appreciated the structure of the intervention package especially since it gradually builds on simple knowledge over time and allows space for paraprofessionals to build relationships with participants:

“*With just two sessions*,* you can’t build a strong relationship with participants – you need more sessions. For example*,* by the final session*,* participants mentioned that just as they started to take an interest in these topics*,* the intervention ended. In other programs*,* this large number of sessions isn’t usually available*” – Paraprofessional.

Another mentioned that “*I built a relationship with participants*,* which encouraged them to start engaging. For example*,* there was one participant who did not speak at all in the beginning*,* but by the last session*,* she started to actively participate and share her thoughts*” - Paraprofessional.

Although paraprofessional ratings were mostly positive from participants’ perspectives with some minor exceptions, paraprofessional ratings by psychologists and midwives revealed more frequent critiques. Across all sessions, psychologists and midwives reported negative assessments of paraprofessional performance up to 26% of the time (see supplementary material). Despite the majority of paraprofessional being given positive ratings, poorer ratings were associated with paraprofessional (1) showing nervousness, and not communicating effectively, (2) having difficulty managing off-topic conversations, (3) focusing on one participants and failing to engage others, especially quiet participants, (4) not knowing how to respond to some questions, (5) not validating or showing empathy when needed, and (6) not knowing how to manage children distractions during sessions (see supplementary material).

That said, paraprofessional echoed these findings and suggested additional training and more time allocation for sessions to better cover all needed topics. Although the training they received before the intervention actively simulated intervention settings and centered mainly on preparing them to lead sessions, our results showed that while paraprofessionals succeeded in understanding the material, they had difficulty presenting and communicating it to participants. That said, it may be worth considering an additional element of having paraprofessionals observe a live or pre-recorded session to model ideal session delivery to them.

Finally, paraprofessionals appreciated in-session presence of psychologists and midwives as they perceived them as a source of support, as one of the mentioned:

“*I have regular contact with the specialists and the other paraprofessionals*,* and we discuss issues that come up during the sessions. If I make a mistake in explaining something*,* they correct me*,* and our communications help us improve our performance and develop ourselves” - Paraprofessional*.

In many cases, their presence was pivotal as paraprofessionals may not have the same level of expertise as them to answer all technical questions that they may be asked during the sessions.

### Logistical considerations

Logistically, our findings suggest that flexibility in scheduling participants is critical to ensure engagement and to limit attrition. As one paraprofessional mentioned:

“*we should accommodate participants’ schedules by offering flexible session timings from the start. Some participants attended certain sessions but missed others because the timing wasn’t convenient for them*,* even though there was a possibility of adjusting the timing*” - Paraprofessional.

During the first week of implementation one participant mentioned thatflexibility is key for participation, as stated below:

“*the timing is flexible*,* they ask us when we can attend*,* and if we are unable to make the scheduled time*,* they give us another time that suits us*” – Participant in the experimental group.

Enablers of participation therefore included easier access to sessions and receiving incentives, cultural acceptance, and having logistical support to help with managing their children and household chores. Participants reported needing additional support including vocational training, having access to safe spaces, potentially attending sessions in camps instead of PHCs, needing support in finances and in the basic survival needs, and requiring easier access to knowledge and resources delivered during the program (see supplementary material).

Our evaluation also showed that daily follow-up phone calls are necessary to remind participants of their sessions especially since they are stretched over 8 weeks of implementation, with each participant attending one session per week. Early during the intervention, we learned that without reminders, attrition was likely to increase. The main reason for skipping sessions as reported by participants was needing to be reminded of the sessions by project team.

Other than war-related challenges, which are discussed in subsequent sections, the main logistical challenges included in-session disruptions caused by the presence of children, as described by one paraprofessional:

“*Many young women were uncomfortable when participants brought their children with them…The number of children was overwhelming… even as a facilitator*,* I felt it was necessary to have a room for children*” – Paraprofessional.

### Contextual considerations

War challenges were perhaps the most important contextual challenges, as one nurse explained:

“*I think what hindered the project was the situation we went through*,* namely war*,* which we hope never happens again. I believe that was the main obstacle. Team members might have been coming from Beirut*,* but due to the war*,* they could no longer attend. Even among refugee participants we had*,* some were displaced and were afraid*,* which may have prevented them from attending*” – Nurse at the PHC.

Indeed, the war posed major risks on the project, however the team adapted by revising implementation plans and handing over logistical considerations to trained local coordinators and research assistants, while still virtually attending each session and conducting daily follow-ups online. This is because the team was based in the capital in Beirut and was not able to travel back and forth to operation sites given consistent air strikes across the country.

While handing over the project to local communities, the team ensured that sites of operations were not under threat of strikes and the safety of participants and field staff were not at risk. Several days were also rescheduled when there were potential risks. As a result of the war, attrition was at 30% by the end of the intervention driven by multiple factors including further displacement, security concerns. As described by a PHC manager:

“*I remember you paused for a while because of the war*,* right? It was like people thought the project was canceled and would no longer take place. So*,* they stopped coming*,* and some people went in different directions – some returned to Syria*,* others moved from one area to another.”* – Head of PHC.

### Content considerations

In general, the content of the intervention was reported to be very relevant to participants’ experiences. One LWC member mentioned that:

“*because the topics are related to mental health*,* family planning*,* and reproductive health*,* these are important for young women who marry at an early age*,* especially as issues like abuse and frequent divorce are prevalent*” – Member of Local Women’s Committee.

For many, these topics were new to them, as reported by one participant “*The topics raised were new to us and we were not aware of them before*” – Participant in the experimental group.

As for the modality, project stakeholders appreciated the fact that it was led by other members of the community that are capable of adequately relating to participants and communicating thisknowledge with them. A PHC manager mentioned that:

“*the presence of people from the same environment as the target group to provide information played a role in making participants feel comfortable” – Head of PHC*.

Another participant mentioned that “*they communicated knowledge in a way that suited us*” – Participant in the experimental group.

Indeed, this was also supported by a paraprofessional “*This is because they are not afraid*,* as the people leading the sessions come from their own community. This influence is very powerful and strengthens [the program]”* – Paraprofessional.

Also, another strong point of the intervention modality was the fact that teaching aids were used as described by an LWC member:

“*when we are on the field*,* with tools available*,* and participants can see samples and examples of what is being explained*,* it becomes easier than just listening to the explanation. This helps them understand better*.” – Member of Local Women’s Committee.

Another participant mentioned that “*they showed us samples of IUDs. It would be great if they showed us samples of more family planning methods to understand more*,* and do more activities regarding assertive communication*,* problem solving*,* and to go deeper into these topics*” – Participant in the experimental group.

Furthermore, one nurse highlighted the importance of integrating psychosocial support with family planning and reproductive health topics:

“*for example*,* a girl at this age may be forced by her parents into early marriage*,* or she may not want children now*,* but they force her to do so. So*,* the psychological burden may manifest as physical pain… that’s when we realized that they complement each other*” – Nurse at the PHC.

As for content-related challenges, one paraprofessional critiqued the time distribution for the session topics by reporting that:

“*some sections were allocated 25 minutes*,* but they only required 10 minutes*,* while others were given 10 minutes*,* but they actually needed 30 minutes*” - Paraprofessional.

Similarly, a participant mentioned “*eight sessions weren’t enough to cover everything; we needed more sessions to delve deeper into the provided information*” – Participant in the experimental group.

She suggested that some sessions may need to be expanded on and others to be made more concise. Additional content related challenges are discussed through a cultural lens in the subsequent section.

### Cultural and gender norms considerations

Despite pervasive cultural beliefs limiting discussions around topics of sexuality and mental health, one participant mentioned that:

“*there has been awareness raised about reproductive and mental health issues. People have become curious. It’s now normal for someone to know these things*,* and cultural barriers are no longer an obstacle*” – Participant in the experimental group.

This was mainly attributed to the fact thatknowledge shared was done through a scientific lens as described by another participant:

“*because they speak scientifically*,* why should anyone be ashamed of knowledge*?” – Participant in the experimental group.

This was also echoed by a nurse at the PHC stating that:

“*it is no longer considered shameful to talk about having depression or other mental health conditions. It is no longer a taboo to go to a pharmacy and ask for antidepressants or to educate women and girls about reproductive health*” – Nurse at the PHC.

Despite this progress in targeted communities, there still remained some cultural barriers that may have affected attendance to, engagement with, and application of SRH, FP, and mental health lessons. For instance, one LWC mentioned that:

“*there were some young women whose spouses might prevent them from attending*” – Member of Local Women’s Committee.

Anotherparticipant mentioned that:

“*some people don’t allow their daughters toparticipate in such a project*.” – Participant in the experimental group.

In-session, cultural resistance to FP and SRH information was also remarked by a paraprofessional:

“*when we were explaining*,* there were groups that were more receptive than others. For instance*,* when we brought out the male genital model and the condom*,* there was a lot of rejection. Later. I decided that if I found a group that would be more accepting*,* then we would conduct the activity. Otherwise*,* we would explain using pictures instead*” - Paraprofessional.

This may be attributed, according to a PHC manager to the fact that:

“*family planning topics contradict their traditions and values*” – Head of PHC.

This aligns with field observations from paraprofessionals, who described how social expectations often constrained girls’ autonomy:

“*for them the girl is obliged to work*,* get married*,* and have children. They believe she does not need anything else… how can we convince them of something they are not accustomed to?*” - Paraprofessional.

Another mentioned that “*when we talked about early marriage*,* the mother or young women would tell us “it is not in our hand”. The community*,* father*,* or brother need to be convinced first before we can do that.*” – Paraprofessional.

However, despite that, to some extent, attending SEEK seemed to have received some approval from most household members as observed by an LWC member:

“*participating girls were engaged*,* and their families and husbands were sending them to attend. If there was anything against the project*,* they wouldn’t have sent them*” – Member of Local Women’s Committee.

To a large extent, these findings highlight the fact that to improve outcomes following this intervention, men may need to be involved throughout implementation. One PHC manager remarked:

“*the project should also target men. Both spouses should be targeted…because men are those who take decisions on such things [family planning]*” – Head of PHC.

This was also echoed by a nurse at the PHC “*you need both parties to be involved to achieve better results. If you explain something to the women… and she gets convinced and goes home*,* but the man is not convinced – for example*,* if she has vaginitis and tells him that it is not advisable to have sexual intercourse and she’s undergoing treatment – do you think he will believe her? Unfortunately*,* in our society*,* there is still a patriarchal mindset. That’s why we need to engage both parties together*” – Nurse at the PHC.

### Additional field observations

In addition to the diverse aforementioned findings, the program’s field team gathered additionalobservations from the field aiming to assess the role of each stakeholder in the program include implementing teams.

#### Psychologists and midwives

This group was essential to ensure a professional outlook on sessions including providing expert answers on technical questions related to SRH or mental health that may be difficult for paraprofessional to answer correctly. This was commonly observed across sessions, and given the scope of the program, this group proved to be pivotal for quality assurance. Additionally, this group, given their certification and expertise in SRH and mental health, contributed another layer of quality assurance beyond ensuring accurate delivery of information, and this included managing difficult situations in-sessions such as when participants displayed signs of distress and anxiety. To a large extent, many participants experienced negative emotions when discussing sensitive topics, and the psychologists intervened, when necessary, privately to support with emotional regulation, or to refer participants who may need additional psychological support or access to protection services.

#### LWCs (Local women Committee)

LWCs were observed to be primarily important communication mediators between the research and implementation teams, enrolled participants, and paraprofessional. Ultimately, LWCs played a mediating role by being fully immersed in the program throughout the process of preparation, training delivery, package implementation, and program evaluation. Their importance lay in their deep understanding of the research and implementation process, as well as their strong roots in the communities being served. Through the process of conducting this research, LWCs facilitated communication with participants during data collection and recruitment, were trained on package material during the training sessions for paraprofessionals, actively contributed during in-session delivery to explain ideas, participate in activities, and support with logistics, and provided their feedback on the package during evaluation. Most importantly, the active presence of LWCs as mediators provided an additional layer of trust and acceptance between enrolled participants and the implementing research team.

#### Research assistants RAs

RAs were primarily responsible for managing participant attendance and participation during data collection and during intervention delivery. As such, throughout the intervention process, RAs learned that participants require consistent reminders to attend the sessions including on the same day of the session. RA’s role in the program was essential to ensure that all participants are continuously communicated with and attended to optimize engagement and minimize attrition. In almost every session, RAs managed tardiness, no-show, and various coordination challenges including re-scheduling participants, and allocating them to different session times, among others.

#### Field coordinators

Each site was assigned a field coordinator to manage the overall process including oversight of staff, enrolled participants, coordination with PHC, and coordination with the research team. In general, their presence was important to ensure that all steps are moving according to plan and that any potential emerging issues related to logistics, coordination, performance, or others, are effectively and efficiently responded to.

Furthermore, additional field observations included an overview of logistical challenges associated with intervention delivery. The first group of challenges included distractions by children either due to their presence in the session, or due to participants having to manage them outside of sessions that tackled sensitive topics where children were not allowed to be present. Based on field notes, almost every session was delayed to start due to late arrivals, required extension due to time management issues by paraprofessionals, and rescheduling/relocation of participants across groups was very common. In addition, paraprofessionals commonly needed more time to explain certain concepts that were deemed difficult by some participants, especially those who were illiterate. In this regard, another observation highlighted some difficulties faced by some participants who could not read/write/or draw, that which limited their engagement relative to their peers.

## Discussion

To our knowledge this is the first study reporting a process evaluation of a community-based intervention conducted through an integrated approach to improve family planning, SRH, and mental health among Syrian refugee women and girls in Lebanon. Community-based interventions are important when working with refugees on complex, sensitive, and culturally nuanced topics, especially when they are designed to be low-resource and low-intensity, and led by community health workers. This is because involving community health workers – or paraprofessionals – in leadership roles may reduce resistance by other community members, increase trust and credibility, ensure more effective communication, and improve sustainability of outcomes [50–52].

In addition, this is one of very few such studies conducted during a period of active conflict in Lebanon. Although during the design, development, and preparation stages, this research did not account for potential of active conflict, war escalated in Lebanon during the first week of field implementation between September and December 2024. Therefore, this study provides added value to the literature by virtue of its design, but also because of contextual changes that required rapid and flexible adaptation plans.

In general, our results show high satisfaction among participants, which also include positive qualitative responses from them, such as them gaining new knowledge, especially on taboo and sensitive topics that they were previously unfamiliar with. However, participants remarked some challenges with the sessions they attended such as being distracted by children accompanying their mothers, perceiving some facilitators as not being clear, and desiring different time structures for the intervention. Importantly, they remarked that knowledge gains and material/monetary incentives were primary drivers of attendance, and their absence would constitute major barriers to attendance. These findings echoe those reported in the Amenah early marriage pilot intervention among Syrian refugees in Lebanon, in that absence of transportation or transportation fee coverage was noted as a potential barrier, likely reducing attendance [53].

Paraprofessionals were also satisfied with the preparatory training, program structure, and content relevance, and they noted that the gradual progression of topics and their ability to build rapport with participants over time was an important aspect. Indeed, designing this low-resource and low-intensity community-based intervention was primarily aimed at ensuring good relationships between paraprofessionals and participants, and the data suggests that this appeared to be a success factor to ensure engagement and effective delivery. Considerable evidence confirms that community health workers ability to support vulnerable communities in accessing care is strongly associated with the trusting relationships they build with community members, which reinforces the importance of this approach [54, 55]. However, there were key gaps in performance as rated and reported by psychologists and midwives attending the sessions, which included difficulties managing discussions and challenging questions, sustaining engagement, and sub-optimal communication. In fact, even paraprofessionals suggested the desire for more preparatory practice sessions. These findings corroborate some of the participants comments above and other findings in the literature and suggest that additional preparatory training may be needed for paraprofessionals specifically on soft skills and communication and presentation skills [56].

At the logistical level, our findings suggest that flexibility is key for engagement and to reduce participant attrition. Flexibility was mainly discussed in the context of scheduling and rescheduling sessions, re-allocation of participant groups, and the need for consistent follow-ups and reminders. This is especially important because refugee women and girls, especially during periods of crisis, as was the case in our study, may have difficulty abiding consistently by set schedules for a multitude of reasons including household responsibilities, transportation difficulties, and child care among others [57, 58]. Indeed, this was problematic and it suggests that having dedicated spaces and staff for childcare is crucial when implementing such a community-based intervention because refugee women may need additional child support to be able to attend. This point was echoed in a UN women report, which states that a lack of accessible childcare services limits women’s mobility [60]. Similar barriers to consistent participation have been documented in other humanitarian settings. For instance, in an in-person community-based psychosocial intervention conducted in Colombia, many women reported missing sessions due to competing domestic responsibilities, including household chores and childcare [59]. In addition, similar findings have been reported regarding the need for regular follow-ups by Buitrago et al., where regular follow-up through phone calls or text messages was essential to foster engagement and maintain participant’s motivation [59]. Finally, Syrian refugees in Lebanon largely depend on humanitarian aid to access basic survival needs [15], and so to ensure engagement and participation, providing monetary incentives is crucial for initial buy-in and to facilitate attendance.

One of the main contextual challenges was associated with war and related factors [61]. By the first week of implementation, the country became bombarded with active strikes, and this prompted the team to quickly pivot implementation plans to account for staff and participant safety, but also to be able to continue delivery of the intervention. The war caused extensive destruction in southern Lebanon, the Bekaa region, and Beirut’s southern suburbs, resulting in high civilian casualties and damage to residential, healthcare, and infrastructure systems, contributing to a growing humanitarian crisis [61]. A major part of this crisis included increased internal displacement for both host communities and refugees alike. In fact, many of the displaced Syrian refugees residing in Lebanon were forced to flee back to their homes and neighboring countries again due to escalating conflict [33].

As for the intervention’s content, participants described it as being new, important, and relevant to their needs, especially given how they were tailored to their contexts. This aligns with evidence indicating that interventions tailored to the cultural and contextual realities of participants are more likely to be accepted and to reach a wider audience [62]. Importantly, because facilitators were from the same communities, their familiarity with local contexts and cultural norms played a key role in addressing participants’s needs effectively [63]. A systematic review of SRH interventions in conflict settings found that engaging community members and leaders in intervention delivery enhances acceptability and ensures that programs are context-specific and culturally appropriate [62].

As for the intervention’s modality, especially when delivered to highly vulnerable groups who are likely to include persons with varying literacy levels, it may require alternative preparation of material that utilize additional visual aids and tools to facilitate communication for those who cannot read, write, or draw [64]. This is important for in-session engagement, but also for take-home material that may be shared with the community at large, and it may include using digital transformation tools. Consistent with this, a randomized trial among married illiterate women in Turkey found that family planning education tailored to participants’ literacy levels and supported by visual materials significantly improved attitudes toward family planning [65]. Another issue was reported to be the need to expand the length of some sessions, as participant described wanting more time to delve deeper into certain topics. Similar issues were noted in the Amenah Early Marriage pilot intervention, where activities took longer than planned [53].

Finally, at the cultural level, evaluation findings suggest that despite pervasive cultural sensitivities around SRH, mental health, and FP topics as being taboo, especially among refugee communities [66–68], this intervention contributed to ongoing momentum to raise awareness and knowledge about these topics. Similar patterns of knowledge improvement have been documented elsewhere. For example, in Sudan, a community-based family planning education program increased the proportion of married women knowledgeable about FP methods from 40% before the intervention to 85% afterward [69]. Likewise, in Ethiopia, women in the intervention group achieved a significantly higher mean knowledge score compared to those in the control group [70].

Despite some progress at the participant level, there were still remaining cultural barriers and resistance around these topics associated with the wider community in which participants live, specifically in terms of male partners, fathers, and other family members. This meant that it may not be enough to only work with girls and women, but there is rather a need to also include the wider family in the process [10, 71]. Among Rohingya refugees in Bangladesh for example, a study found that husband disapproval was a leading reason for non-use of contraceptives [75], underscoring the influence of male partners in reproductive decision-making and the importance of engaging men in SRH interventions. Evidence supports this approach, showing that male involvement in FP education can play a key role in increasing acceptance, intention, and sustained use of contraceptives, particularly where cultural norms encourage high fertility [72, 73].Supporting this, a scoping review in Sub-Saharan Africa found that involving men in reproductive health programs is practical and effective, increasing family planning uptake, improving partner communication, and challenging harmful gender norms [74].

### Limitations

It is important to recognize some pertinent limitations of this study. For instance, this includes social desirability bias, especially when considering self-reported satisfaction or perceived benefits. It is possible that participants and staff may have been inclined to positively evaluate the intervention to ensure positive relationships with study teams and therefore to not compromise any potential benefits they may receive from future initiatives. However, participants were informed at all data collection time periods that this would not be the case, and that their participation in data collection and regardless of whether or not their responses are positive or negative, would not impact their chances of receiving any services they may be otherwise eligible for. Another important limitation is the fact that the study was conducted during a period of active conflict which was not originally accounted for as a potential risk for the study. For instance, attrition rose up to 30% by the end of the intervention, which is still considered acceptable given the larger context in which it was implemented. Nevertheless, implementing study under different circumstances may have yielded different results. Finally, given the nature of the study design, namely with non-married women being ineligible for participation, this may have introduced some selection biases. However, this was an essential step in this study because in that particular culture, non-married women are expected not to be sexually active, and the topic of sexuality is very sensitive and taboo which makes it difficult to have these conversations with non-married women. In this regard, exclusion of non-married women from participation was conducted to ensure cultural sensitivity.

## Conclusion

In conclusion, this process evaluation suggests that SEEK, as a low-resource and low-intensity community-based intervention, can be effectively implemented in complex settings, including periods of active conflict, because it is designed to be led by members of targeted affected communities. Particularly, SEEK’s design was especially useful to deliver topics that are culturally-sensitive and complex to address such as SRH, family planning, and mental health. This is because the intervention capitalized on active involvement of trained paraprofessionals who were found to be ideal facilitators due to their identities aligning with those of refugee communities.

Also, an important takeaway is that flexibility is key when implementing such an intervention in a highly volatile and unstable context, especially during periods of on-going war. This includes not only flexibility in re-scheduling and timing of sessions, but also ensuring flexibility through recruitment and preparation of field teams. Although it was not part of the original plan to involve trained research assistants from affected communities to lead on field coordination, the situation demanded such a swift response to complete the intervention.

Despite the success of this intervention, and the strong evidence supporting its feasibility and acceptability, including during active conflict, many challenges were experienced and they are discussed above. Subsequent efforts should account for these lessons learned and recommendations to optimize such interventions. Finally, this study found that affected communities, especially during periods of active conflict, can and should be play a central not only at the planning and findings validation stages, but also during implementation and coordination as part of the core research team.

**Table 3:** Post session participant satisfaction survey

Questions	Session rating	1	2	3	4	5	6	7	8
The quality of the session	Excellent	133(78.2%)	140(87%)	103(78.6%)	105(86.1%)	119(94.4%)	107(87%)	112(91.8%)	115(94.3%)
	Good	31(18.2%)	21(13%)	28(21.4%)	17(13.9%)	7(5.6%)	16(13%)	10(8.2%)	7(5.7%)
	Fair	4(2.4%)	0	0	0	0	0	0	0
	Poor	2(1.2%)	0	0	0	0	0	0	0
The session helped you to handle your problems effectively	Yes, it will help a great deal	122(71.7%)	126(78.3%)	110(84%)	107(87.7%)	114(90.5%)	108(87.8%)	104(85.2%)	115(94.3%)
	Yes, it will help somewhat	46(27.1%)	34(21.1%)	21(16%)	15(12.3%)	12(9.5%)	15(12.2%)	18(14.8%)	7(5.7%)
	No, it didn’t help	2(1.2%)	1(0.6%)	0	0	0	0	0	0
Overall satisfaction with the session	Very satisfied	143(84.1%)	131(81.4%)	113(86.3%)11	112(91.8%)	115(91.3%)	111 (90.2%)	112 (91.8%)	117(95.9%)
	Mostly satisfied	25(14.7%)	30 (18.6%)	18 (13.7%)	10(8.2%)	11(8.7%)	11(9%)	10(8.2%)	5(4.1%)
	Indifferent or mildly dissatisfied	2(1.2%)	0	0	0	0	0	0	0
	Quite dissatisfied	0	0	0	0	0	1(0.8%)	0	0

**Table 4:** Post session participant satisfaction survey (Qualitative data)

Session number Feedback	Session 1	Session 2	Session 3	Session 4	Session 5	Session 6	Session 7	Session 8
Feeling of positive emotions (comfort, enjoyment, gratitude)	X	X	X	X	X	X	X	X
Finding the facilitator unclear and unengaging	X	_	_	_	_	X	_	X
Distraction due to the presence of children during the session	X	X	_	_	_	_	_	X
Wishing to adhere to the set time	X	X	_	_	_	_	_	_
Wishing for a longer session duration	X	_	_	_	_	_	_	X
Provision of knowledge and skills for personal development	X	X	X	X	X	X	X	X
Observing improvement in session quality over time	_	X	_	_	_	_	_	_
Anticipation of future and more sessions	_	X	_	_	_	_	X	_
Incorporating videos into the sessions	_	_	_	_	_	_	_	X

“X” refers to whether the given feedback was mentioned in narrative report from participants in a given session

**Table 5:** Participants package satisfaction survey

Indicator				
	**Poor**	**Fair**	**Good**	**Excellent**
Quality of Service received with SEEK program	0	2 (1.6%)	8 (6.3%)	116 (92.1%)
	**No definitely not**	**No not Really**	**Yes Generally**	**Yes Definitely**
Receiving the wanted Service with SEEK program	0	1 (0.8%)	25 (19.8%)	100 (79.4%)
	**None of my needs have been met**	**Only a Few of my need have been met**	**Most of my Needs have been met**	**Almost All of my needs have been met**
Extent SEEK met your need	0	1 (0.8%)	21 (16.7%)	104 (82.5%)
	**No**,** definitely not**	**No**,** I don’t think so**	**Yes**,** I think so**	**Yes**,** definitely**
Recommend SEEK Program to friend	0	1 (0.8%)	3 (2.4%)	122 (96.8%)
	**Quite Dissatisfied**	**Indifferent or mildly satisfied**	**Mostly Satisfied**	**Very Satisfied**
Satisfaction with Help received	1 (0.8)	0	12 (9.5%)	113 (89.7%)
	**No**,** make things worse**	**No**,** really didn’t help**	**Yes**,** helped somewhat**	**Yes**,** helped great deal**
Received Service Helped you dealing with your problems	0	0	7 (5.6%)	119 (94.4%)
	**Quite dissatisfied**	**Indifferent or mildly dissatisfied**	**Mostly Satisfied**	**Very Satisfied**
Overall Satisfaction with Service Received with SEEK program	0	0	1 (0.8%)	125 (99.2%)
	**No**,** definitely not**	**No**,** I don’t think so**	**Yes**,** I think so**	**Yes Definitely**
Seeking Similar Programs	0	0	5 (4%)	121 (96%)
PART II: Program content/Setting/Facilitator				
	**Completely Disagree**	**Neutral**	**Partially Agree**	**Completely Agree**
Relevance of Information to young women	0	0	3 (2.4%)	123(97.6%)
Confidence About Information Accuracy	0	0	5 (4%)	121 (96%)
Learning New health information	0	0	0	126 (100%)
Feeling of Better Ability to deal with problems	0	2 (1.6%)	16 (12.7%)	108 (85.7%)
Convenient Program Location	1 (0.8%)	1 (0.8%)	4 (3.2%)	120 (95.2%)
Safe Program Location	0	0	1 (0.8%)	125 (99.2%)
Warm and Understanding facilitator	0	3 (2.4%)	4 (3.2%)	119 (94.4%)
Competent and Well-trained facilitator	0	4 (3.2%)	3 (2.4%)	119 (94.4%)
Knowledgeable facilitator about SEEK information	0	5 (4%)	3 (2.4%)	118 (93.6%)
Well Organized facilitator	0	5 (4%)	1 (0.8%)	120 (95.2%)

## Data Availability

The datasets generated and/or analyzed during the current study are available from the corresponding author on reasonable request.
